# Comparison of microscopic and endoscopic views in cadaveric ears

**DOI:** 10.1007/s00405-020-05900-4

**Published:** 2020-03-14

**Authors:** John Guy, Jameel Muzaffar, Christopher Coulson

**Affiliations:** 1 Medical School, College of Medical and Dental Sciences, University of Birmingham, Edgbaston, Birmingham, B15 2SG UK; 2 Department of Otolaryngology, Queen Elizabeth Hospital Birmingham, Edgbaston, Birmingham, B15 2TH UK

**Keywords:** Otology, Endoscopy, Microsurgery, Tympanic membrane

## Abstract

**Purpose:**

The advent of endoscopic otosurgery provides reduced tissue destruction with theoretically improved views, yet a quantification of the difference of exposure between microscopic and endoscopic approaches has not yet been performed in human specimens. The objective of this study was to assess the difference in views of cadaveric tympanic membranes when inspected with operating microscopes or endoscopes.

**Methods:**

A circular graduated disc was inserted into eight cadaveric external ear canals to rest against the tympanic membrane. Three independent observers assessed the maximum possible observable radius of the graduated disc in each ear using a 0° endoscope and a surgical microscope in superior, inferior, posterior, and anterior directions.

**Results:**

The endoscope was able to view a significantly larger mean maximum visible radius than the microscope in posterior, superior, anterior, and inferior directions. This represented a mean gain in observable distance of 19.18%. There was a smaller variation in mean maximum visible radius than the microscope.

**Conclusion:**

The wider field of view in an endoscope compared to a microscope implies reduced surgical tissue damage is needed to provide sufficient operative exposure. Enhanced views of the attic were demonstrated by the endoscope, further indicating utility in cholesteatoma observation and surgery.

**Electronic supplementary material:**

The online version of this article (10.1007/s00405-020-05900-4) contains supplementary material, which is available to authorized users.

## Introduction

A major dichotomy in operative surgery is that safe practice requires direct vision of structures, yet in providing appropriate access to maximise the observable field, healthy tissue is often removed. Operating microscopes have been conventionally used in otological surgery since the 1950′s, yet are restricted by predefined sight lines and the anatomy of the external ear canal [1]. This leads to areas of the middle ear which cannot be directly visualised without removal of normal tissue [2].

Harold Hopkins designed the Hopkins Rod rigid endoscope in 1966 [3]. The array of lenses and glass rods effectively transports the user’s observation point from the eye piece to the tip of the endoscope, closer to the target under observation. This delivers high-quality imaging without the need for further magnification while increasing the field of view as there is no encroachment from the canal. It is, therefore, intuitive that it will be of use in difficult to access spaces. First used for otological applications in 1967, the role of the endoscope has expanded from an aid to diagnosis to use as the sole visual device in otological operations [4–6]. The ability to survey the surgical field from within allows for a wider circumference of view and more comprehensive surveillance with decreased invasiveness, at the expense of one-handed surgery and loss of depth perception.

Endoscopy has been shown to improve outcomes in some otological operations, from reduced recurrence of cholesteatoma to more effective tympanoplasty [7–9]. A recent systematic review and meta-analysis highlighted lower canaloplasty rates, better cosmetic results and shorter operating times for endoscopic vs microscopic tympanoplasty, and reduced pain and risk of chorda tympani injury for patients undergoing stapes surgery [10].

Previous studies have examined the difference in the field of view provided by endoscopes and microscopes, from computer modelling to dry skeletal models, and have demonstrated the advantages of the endoscope at viewing the internal spaces of the middle ear [11, 12]. However, quantitative evidence of the increase in field of view afforded by endoscopes in real anatomy has not yet been shown in the literature. Limited research exists to describe the differences of the view of the mesotympanum, an area in which the microscope is often employed. To assess these two methods of observing the ear, we compared the difference in view of cadaveric tympanic membranes when inspected with operating microscopes and endoscopes.

## Materials and methods

**Fig. 1:**
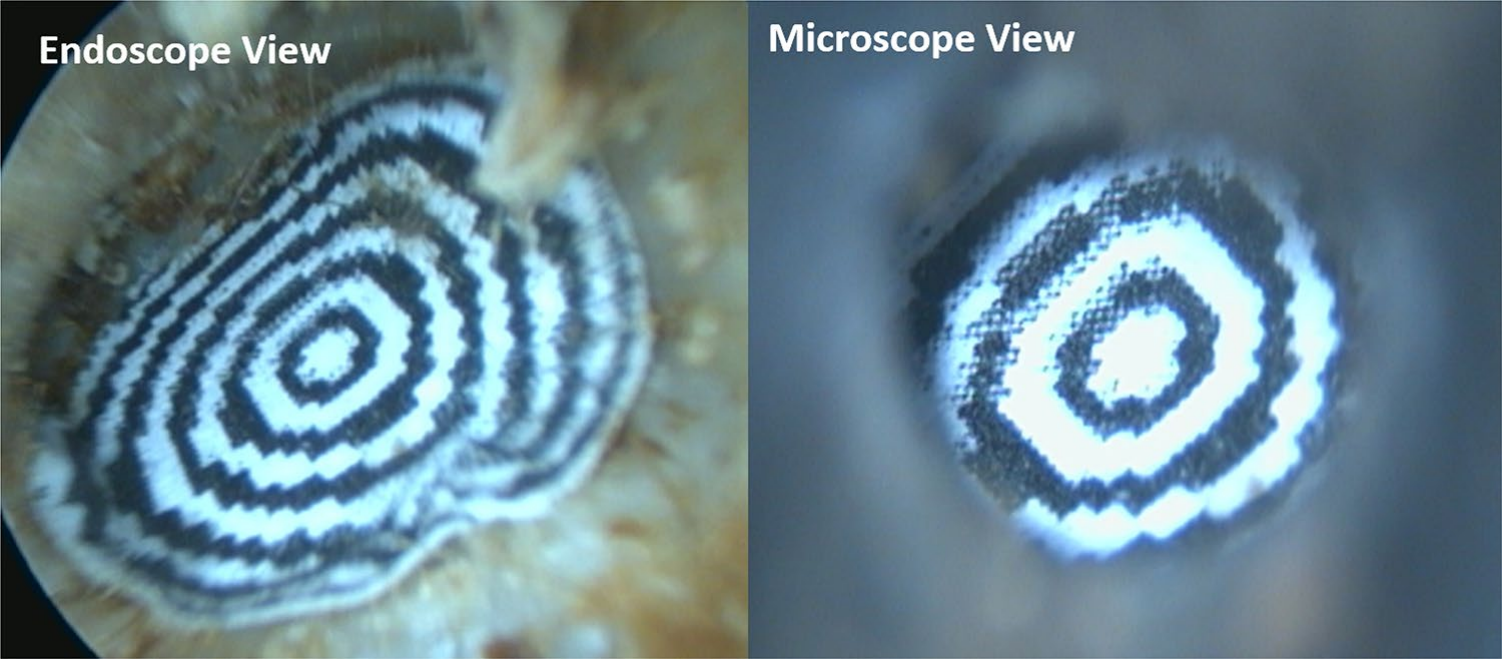
Circular graduated discs in situ

Four formalin-fixed cadaveric heads were used in this study, providing a total of eight ears. The ears were dewaxed until the observational route was clear, and a circular graduated disc gauged at 1 mm intervals was inserted into the acoustic canal and laid flat against the tympanic membrane. Once in place, observations were taken with a 0°—18 cm Hopkins rod connected to a high-definition camera (KARL STORZ GmbH & Co. KG, Tuttlingen, Germany) and an optical microscope (DP Medical, London, United Kingdom). The instruments were permitted to pivot their field of view around the ear to obtain the most optimal view, attempting to observe the most distal ring possible. The maximum visible radius (MVR), defined as the most distal observed ring, was measured in the anterior, superior, posterior, and inferior planes, and was recorded for both instruments in all eight ears. Three observers repeated this process for each ear with the microscope. The mean MVR that could be observed with the microscope and endoscope in all four planes of measurement were calculated for each ear. Figure 1 demonstrates the circular graduated discs in position. The proportional difference in MVR between endoscope and microscope was calculated. Microsoft Excel 2019 was used to analyse these data. All cadaveric imagery was derived from individuals who had previously consented for image distribution, and no identifiable information was included with the images.

## Results

Using the above methodology, we compared the mean maximal visible radius (MVR) across all eight ears between microscope and endoscope. Tables 1 and 2 display the aggregate MVR scores across all specimens for microscope and endoscope. Table 3 shows the results of a two-tailed Student’s *T *test at *p* > 0.05 used to assess statistical significance.

**Table 1:** MVR per sample observed by an operating microscope

Microscope	A	B	C	D	E	F	G	H	Mean	SD	CI
Posterior	11.0	12.3	12.3	14.0	12.0	14.0	14.0	15.0	13.08	1.35401	1.13198
Superior	12.3	13.0	11.7	14.7	12.0	14.3	14.0	14.0	13.25	1.15126	0.96248
Anterior	10.3	10.3	9.0	8.3	11.3	11.3	10.3	10.3	10.17	1.03892	0.86856
Inferior	9.0	10.0	10.3	11.3	12.0	11.0	13.3	11.3	11.04	1.31460	1.09903

**Table 2:** MVR per sample observed by an otological endoscope

Endoscope	A	B	C	D	E	F	G	H	Mean	SD	CI
Posterior	14.0	14.0	14.0	15.0	15.0	15.0	14.0	15.0	14.50	0.53452	0.44687
Superior	14.0	14.0	14.0	15.0	15.0	15.0	14.0	15.0	14.50	0.53452	0.44687
Anterior	13.0	14.0	13.0	15.0	15.0	15.0	11.0	15.0	13.88	1.45774	1.21870
Inferior	12.0	12.0	13.0	11.0	15.0	15.0	14.0	14.0	13.25	1.48805	1.24404

**Table 3:** Comparison of differences observed between endoscope and microscope with *p* value calculation

E–M	Mean	% Change	SD	SEM	CI	*p*
Posterior	+ 1.42	+ 10.83	1.16496	0.41188	0.97393	0.01084
Superior	+ 1.25	+ 9.43	1.01965	0.36050	0.85245	0.01044
Anterior	+ 3.71	+ 36.48	1.69441	0.59907	1.41656	0.00045
Inferior	+ 2.21	+ 20.00	1.40224	0.49577	1.17230	0.00296
Mean % Change		+ 19.18				

The endoscope was able to view a significantly larger mean MVR than the microscope in all four directions. It represented a mean gain in observable distance across all directions of 19.18%.

The endoscope outperformed the microscope in all directions in terms of mean MVR, but showed a markedly larger mean MVR in the anterior (36.48 ± 1.69%) direction, which was statistically highly significant. A larger variance in results was seen across the microscope readings than from the endoscope.

## Discussion

Approaches to the middle ear are dictated by the need to provide an appropriate view while limiting tissue damage. Transcanal approaches remain the least invasive method of access, yet microscopic viewing options face difficulty due to the curvature of the ear canal hampering a wide view of the tympanic membrane and middle ear. The wide field of view afforded by an endoscope allows users to ‘look around corners’ and assess areas of the middle ear that remain off limits to traditional microscopy, such as the anterior recess, attic and the retrotympanum, relegating the microscope to mesotympanic operations. This study has shown that endoscopic transcanal approaches in vivo provide nearly 20% more visibility of the tympanic membrane than the microscopic view, even after the best possible views of each area were assessed. The endoscope was of particular advantage anteriorly, due to the temporomandibular joint and the overlying anterior canal wall impacting the straight line (microscopic) view from the meatus to the anterior recess [13]. There was a significant improvement in field of view superiorly by the endoscope, with less variance across both modalities. This implies the clinical advantage of the endoscope in assessing attic lesions. This is backed up by an assessment of the anatomy, where a direct line passes from the external acoustic meatus to the attic [6]. The results of this study add further weight to the advantage of endoscopic approaches to cholesteatoma.

While a good view is key to good surgery, there are limitations to otoendoscopy. A steep learning curve and the requirement of one-handed surgery can limit its effectiveness in certain procedures [14]. This experiment also faced several limitations. The use of cadaveric specimens was intended to give a good representation of operative anatomy, yet the ears used were of modest quality. Debris was prevalent throughout the external canals, which were themselves narrower and less pliable than in vivo specimens, limiting the clinical similarity of this result. The graduated discs used were friable and difficult to place, meaning that there was wide variance in how closely they lay against the tympanic membrane. There was limited formal centralisation of the graduated discs, and the material was prone to buckle and shift in position, meaning that the centre of the radial measurements was not kept constant between specimens. The lack of a video output from the microscope also contributed to variance of those results, as each assessor had to make their own judgement of the maximum visible radius.

Further studies in assessing the field of view could also be undertaken in the middle ear itself. Assessment of the view of important structures could be implemented using this scaled graduated disc system, exploring the degree that the sinus tympani or other recesses can be observed.

## Conclusion

In this study, endoscopy was shown to provide a greater view of the tympanic membrane than microscopy. This greater field of view may assist the surgeon as they translate their anatomical knowledge into clinical practice. This may help to explain the shorter operative times seen in published studies for endoscopic tympanoplasty despite the limitations of operating one handed.

## Electronic supplementary material

Below is the link to the electronic supplementary material.

Supplementary file1 (XLSX 55 kb)
